# Basic Helix-Loop-Helix Transcription Factor Bmsage Is Involved in Regulation of *fibroin H-chain* Gene via Interaction with SGF1 in *Bombyx mori*

**DOI:** 10.1371/journal.pone.0094091

**Published:** 2014-04-16

**Authors:** Xiao-Ming Zhao, Chun Liu, Qiong-Yan Li, Wen-Bo Hu, Meng-Ting Zhou, Hong-Yi Nie, Yin-Xia Zhang, Zhang-Chuan Peng, Ping Zhao, Qing-You Xia

**Affiliations:** 1 State Key Laboratory of Silkworm Genome Biology, Southwest University, Chongqing, China; 2 Key Sericultural Laboratory of the Ministry of Agriculture, College of Bio-Technology, Southwest University, Chongqing, China; U. Kentucky, United States of America

## Abstract

Silk glands are specialized in the synthesis of several secretory proteins. Expression of genes encoding the silk proteins in *Bombyx mori* silk glands with strict territorial and developmental specificities is regulated by many transcription factors. In this study, we have characterized *B. mori sage*, which is closely related to *sage* in the fruitfly *Drosophila melanogaster*. It is termed *Bmsag*e; it encodes transcription factor Bmsage, which belongs to the Mesp subfamily, containing a basic helix–loop–helix motif. Bmsage transcripts were detected specifically in the silk glands of *B. mori* larvae through RT-PCR analysis. Immunoblotting analysis confirmed the Bmsage protein existed exclusively in *B. mori* middle and posterior silk gland cells. *Bmsage* has a low level of expression in the 4^th^ instar molting stages, which increases gradually in the 5^th^ instar feeding stages and then declines from the wandering to the pupation stages. Quantitative PCR analysis suggested the expression level of *Bmsage* in a high silk strain was higher compared to a lower silk strain on day 3 of the larval 5^th^ instar. Furthermore, far western blotting and co-immunoprecipitation assays showed the Bmsage protein interacted with the fork head transcription factor silk gland factor 1 (SGF1). An electrophoretic mobility shift assay showed the complex of Bmsage and SGF1 proteins bound to the A and B elements in the promoter of *fibroin H-chain* gene*(fib-H)*, respectively. Luciferase reporter gene assays confirmed the complex of Bmsage and SGF1 proteins increased the expression of *fib-H*. Together, these results suggest Bmsage is involved in the regulation of the expression of *fib-H* by being together with SGF1 in *B. mori* PSG cells.

## Introduction

The silkworm, *Bombyx mori*, which is a key economic insect often used as a model for lepidopteran insects, has huge economic value for its potential applications in industry and medical technology [1]–[3], as well as for studying the fundamental mechanisms underlying gene regulation and organ differentiation [4]. The *B. mori* silk gland is the focus of this study because silk production is the major, if not the only, purpose for domestication of this insect. The *B. mori* silk gland is a specifically differentiated silk-producing organ composed of the anterior silk gland (ASG), middle silk gland (MSG) and posterior silk gland (PSG). The ASG is responsible for silk spinning, the MSG secretes sericin and the PSG produces fibroin. The sericin protein is a complex containing at least five sericin proteins encoded mainly by *ser1, ser2* and *ser3* through alternative splicing of mRNA. Each is expressed in certain parts of the MSG during embryonic and larval development [5]. Fibroin is composed of three main protein components, fibroin heavy chain (fib-H), light chain (fib-L) and P25, encoded by the corresponding genes, which forms a hexameric structure with a fib-H/fib-L/P25 ratio of 6∶6∶1 [6]. Among them, the *fib-H* gene is highly expressed in PSG cells but is repressed in MSG cells. The 5′ flanking sequence of *B. mori fib-H*, which is required for maximal transcription in vitro, contains five regions that bind protein factors from the PSG extract [7]. However, details of the mechanism underlying the ability to be synthesized effectively in silk glands and the precise regulation of how *fib-H* is highly expressed in PSG cells but is repressed in MSG cells is not fully understood.

Several transcription factors involved in transcriptional regulation of the *fib-H* have been identified, including *Bombyx* Fkh/SGF-1 [8], which is a homologue of the protein encoded by the *Drosophila* region-specific homeotic gene fork head [9], SGF-2 [10], [11], POU-M1/SGF-3 [12] which is a homologue of *Drosophila* Cf1-a, and FMBP-1 [13], [14]. The gene expression profiles of those factors in the silk glands have been characterized individually [15] or by genome-wide analysis [16], expression of the *fib-H* gene seems to be controlled directly by the co-ordination of these factors, which are expressed differentially during silk gland development. However, whether other factors or partners are involved in the regulation of silk genes needs further study.

A superfamily of transcription factors containing a basic helix–loop–helix domain (bHLH) has important roles in the control of cell proliferation, determination and tissue differentiation during the development of animals and plants [17], [18]. The bHLH domain, which is approximately 60 amino acids in length, comprises a DNA-binding basic region of 15 amino acids residues followed by two α helices separated by a variable loop region [19]. Besides binding to DNA, the bHLH domain also promotes dimerization, allowing the formation of homodimer or heterodimer complexes [20], [21]. Previous studies showed that the cycle homolog (*Bmal1*) belongs to the bHLH-PAS subfamily and is expressed in all silkworm tissues tested [22]. The 52 *bHLH* genes identified in *B. mori* belong to 39 *bHLH* families in groups A–F, respectively [23]. In *D. melanogaster*, sage is a salivary gland-specific bHLH protein that works with Fkh protein to regulate expression of *SG2* directly as well as to express *sage* itself, and Fkh and sage regulate *SG1* indirectly [24]. The salivary glands of *Drosophila* and the silk glands of *B. mori* are likely to be homologous organs [25], [26], and specialized for the massive production of several tissue-specific secretory proteins. Presumably, they might have a similar function in *B. mori* silk glands. In this study, we identified a Mesp subfamily bHLH transcription factor termed *Bmsage* and demonstrated it is expressed specifically in the silk glands of *B. mori*. In addition, we confirmed that the Bmsage protein interacted with a fork head protein (SGF1) and formed a complex that bound to the A and B elements in the promoter of *fib-H* to increase expression of the *fib-H* gene.

## Materials and Methods

### Insects

The wild type strain Dazao (low silk strain which produces little silk proteins) and strain 872 (high silk strain which produces much more silk proteins) were obtained from the Gene Resource Library of Domesticated Silkworm, Southwest University, China. The cocoon shell weight and the cocoon shell rate of strain 872 is higher than strain Dazao. The silkworms were reared on fresh mulberry leaves or on artificial diets at 25°C under a photoperiod of 12 h light/12 h dark with 75% relative humidity.

### Bioinformatics analysis of candidate genes

Scanning and identification of candidate genes were done from the *B. mori* microarray database (http://silkworm.swu.edu.cn/silkdb/) and verified by domain prediction using SMART (http://smart.embl-heidelberg.de/). The prediction of an open reading frame and translation of amino acid sequences were preformed in ExPaSy (http://www.expasy.org/tools/). Phylogenetic analysis was conducted with the candidate gene and that of invertebrate and vertebrate species belonging to the *bHLH* superfamily, including *D. melanogaster*, *Glossina morsitans*, *Aedes aegypti*, *Mus musculus*, *Danio rerio* and *Branchiostoma floridae*. Amino acid sequences of the annotated genes in these species were downloaded from NCBI (Table. S1). Amino acid sequences were aligned with Clustal X using default parameters [27] and the phylogenetic analysis was carried out using a neighbor-joining (NJ) method with programs in MEGA version 5.0 [28].

### RT-PCR and qPCR

Total RNA was prepared using TRIzol reagent according to the manufacturer's protocol. Reverse transcription was carried out with 1 ug of total RNA in 10 μL reactions with a PrimeScript RT reagent Kit (Takara) according to the manufacturer's recommendations. Primer sets for RT-PCR of *Bmsage* is given in Table S2. The semiquantitative RT-PCR conditions were: 94°C for 30 s followed by 28 cycles at 94°C for 10 s, 60°C for 15 s then at 72°C for 90 s. Finally, an elongation step at 72°C for 7 min. The silkworm housekeeping gene encoding ribosomal protein L3 (*BmRpl3*: GenBank accession no. NM_001043661.1) was used as an internal control for normalization of sample loading.

Quantitative PCR (qPCR) was performed with an ABI7500 real-time PCR machine (Applied Biosystems) using FastStart Universal SYBR Green Master (Roche). Each amplification reaction was done in a 15 μL qPCR reaction under the following conditions: denaturation at 95°C for 10 min followed by 40 cycles of treatment at 95°C for 10 s, 60°C for 30 s then at 72°C for 35 s. The gene encoding ribosomal protein L3 (BmRpl3) was used as a control gene. The primers for qPCR reaction are given in Table S2. The threshold cycle of each target product was determined and set in relation to the amplification plot of *BmRpl3*. The detection threshold was set to the log linear range of the amplification curve and kept constant (0.05) for all data analysis. Difference in C_T_ values (ΔC_T_) of two genes was used to calculate the relative expression [29]. The transcript abundance value of each individual was calculated as the mean of three replicates.

### Recombinant expression, purification and western blot

The coding region of *Bmsage* was PCR-amplified using a sense primer (5′-CGCGGATCCATGTACAATCAAACATAC-3′) with an *Bam*HI site upstream of the first amino acid residue and an antisense primer (5′-CCCAAGCTTAGTATCTCTGTTGACGC-3′) with a *Hind*III site downstream of the amino acid residue. The purified PCR product was digested with *Bam*HI/*Hind*III and ligated into pET28a vector (Novagen), resulting in a recombinant expression vector pET-28a/Bmsage. The resultant plasmid was sequenced and transformed into *Escherichia coli* strain BL21 (DE3) competent cells (TransGen) and grown at 37°C in Luria-Bertani medium containing kanamycin (final concentration, 20 μg/ml), then induced with 0.2 mM isopropyl β-D-1-Thiogalactopyranoside (IPTG). Purification of the recombinant Bmsage protein was achieved using Ni-NTA affinity column (GE Healthcare) according to the manufacturer's instructions. Phenylmethylsulfonyl fluoride (PMSF, 100 mM) was used as a proteinase inhibitor (Roche) during the purification. The purified protein was then subjected to the G-25 column (GE Healthcare) for desalting and exchanging the buffer with 20 mM Tris-HCl, 500 mM NaCl, pH 8.0. The separated lysate was detected using SDS-PAGE (15% (w/v) polyacrylamide gel) and the concentrations of proteins were measured using a BCA kit (Beyotime, China). The purified proteins were injected into New Zealand White rabbits for preparation of antibodies.

The Malpighian tubule, fat body, head, midgut, ASG, MSG, PSG, epidermis and gonad were collected from larvae on day 3 of the fifth instar. The tissues were homogenized in 10 mM sodium phosphate-buffered saline (PBS, pH 7.4) containing a mixture of proteinase inhibitors (Roche). The supernatant of homogenates was collected by centrifugation (12 000 g, 4°C, and 10 min) and the protein concentrations were measured using a Bradford Assay Kit (Tiangen, China) with bovine serum albumin (BSA) used as the standard. The proteins were separated by SDS-PAGE (15% (w/v) polyacrylamide gel) and electroporated onto polyvinylidene difluoride (PVDF) membrane (Roche). The membranes were blocked in 5% (v/v) skim milk overnight at 4°C and incubated with a primary antibody for 1 h at 37°C. The membranes were washed, incubated with goat anti-rabbit IgG conjugated with alkaline phosphatase (Sigma) as a secondary antibody for another 1 h at 37°C then visualized using CDP-Star chemiluminescent substrate (Tropix).

### DNA constructs and Cell culture

The promoter of *fib-H* (-865 - +1 bp) (forward primer: 5′-CGGGGTACCAAGCTTGTTGTACAAAACTG-′3; reverse primer: 5′-CTAGCTAGCGCTGATTTGAAAAAGTTGAA-′3, set as described [30]) was cloned into the luciferase reporter plasmid pGL3-fibH-Luc (pGL3 basic vector (Promega) at the N-terminal end) between the *Kpn*I and *Nhe*I restriction sites. Three different 5′-truncated fragments were created by PCR amplification from the pGL3-fibH-Luc reporter plasmid and inserted into pGL3-Basic as described above. For expressing vectors, the primer sequences of *Bmsage* and SGF1 were set as its open reading frame and contained a *Bam*HI and a *Not*I restriction site, respectively. The target fragments were obtained by electrophoresis in a 1% (w/v) agarose gel and then cloned into a *Bam*HI and *Not*I-digested 1180 [Hrs1000-BmAct4-LUC-Ser1PA] expression vector (maintained in our lab). Highly purified plasmid DNA was prepared using the QIAGEN Plasmid Midi Kit (Qiagen, Germany). *B. mori* cell line *Bm*E (developed originally from an embryonic cell) [31], which express endogenous *Bmsage* and SGF1 genes (data not shown), was maintained at 27°C in Grace medium supplemented with 10% fetal bovine serum (HyClone). The transfection vectors were transfected into *Bm*E cells using X-tremeGENE HP DNA Transfection Reagent (Roche) as described by the manufacturer's instructions using the enhanced green fluorescent protein (EGFP) transfection vector as the control. Cotransfection was repeated three times, and the average expression levels of a target gene is represented as mean ± SE.

### Far-western blot, ELISA

To obtain soluble proteins for protein-protein interaction studies, the full-length Bmsage-glutathione *S*-transferase (GST) fusion protein expression plasmid was constructed by subcloning the full-length Bmsage cDNA into the GST gene fusion vector pGEX-4T-1 (Amersham Biosciences) in the correct reading frame between the *Bam*HI and *Not*I restriction sites. Purification of the recombinant Bmsage-GST was performed using GST affinity column (GE Healthcare). The SGF1 coding region was subcloned into pET28a vector using a sense primer (5′-CGCGGATCCATGATCTCGCAGAAGCT-3′) with a *Bam*HI site upstream of the first amino acid residue and an antisense primer (5′-CCGGAATTCTCACAAGGGCGGCTGCG-3′) with an *Eco*RI site downstream of the amino acid residue. Soluble SGF1 was purified by affinity chromatography using Ni-NTA resin.

Far-Western blots were performed as described of Wu et al [32]. The purified protein (1 μg) was separated by SDS-PAGE (15% (w/v) polyacrylamide gel) as prey protein. Following electrophoresis, proteins were transferred to PVDF membranes. The proteins in the membrane were denatured and then renatured. The membrane was blocked and probed with purified bait protein, which was detected by the standard WB method using BSA and GST proteins as negative controls.

To further confirm Bmsage protein interacts with SGF1 protein in vitro, the binding of Bmsage and SGF1 was detected by an enzyme-linked immunosorbent assay (ELISA) [33]. The purified His-SGF1 and BSA (0.5 μg) dissolved in 50 mM Tris-HCl (pH 7.4) containing 12.7 mM EDTA were fixed in a 96-well polystyrene plate overnight at 4°C. The plate was washed three times with PBS (containing 0.05% Tween-20, pH 7.4) and then blocked with 1% (w/v) BSA in PBS for 2 h at 37°C. After three washes with PBS, the plate was incubated with Bmsage-GST in PBS for 2 h at 37°C then probed with rabbit polyclonal antibody against Bmsage and secondary antibody (conjugated with HPR) at 37°C. After three washes with PBS, 3,3′,5,5′-tetramethylbenzidine (Beyotime, China) was added into the plate as chromogenic reagent and kept in darkness for 15 min. The absorption at 450 nm was detected by Microplate Reader (Bio-rad).

### Immunohistochemistry and co-IP assays

Immunohistochemical colocalization of Bmsage and SGF1 was performed as described in Liu et al. [34]. For immunostaining, *Bm*E cells were grown on glass cover slips in the growth medium. Cells were transfected with expression plasmids by using X-tremeGENE HP DNA Transfection Reagent (Roche) as described above. After transfection for 48 h, cells were fixed for 10 min at room temperature with 4% (v/v) formaldehyde in PBS and blocked for 30 min in PBS containing 0.1% (w/v) BSA and 5% (v/v) goat serum. Treatment with the primary antibody [anti-HA monoclonal antibody mouse (Sigma), anti-Flag monoclonal antibody M2 mouse (Sigma), anti-Bmsage antibody rabbit] was for 1 h, followed by incubation with the secondary antibody (anti-rabbit IgG FITC or anti-mouse Alexa 555) for 30 min, both at room temperature. Finally, the samples were mounted using a mounting medium containing 4′,6-diamidino-2-phenylindole (DAPI) and photographed using confocal microscopy (Japan).

To confirm whether Bmsage protein interacts with SGF1 protein in vivo, nuclear extracts were prepared from *Bm*E cells by over-expressing Flag-Bmsage and HA-SGF1. A 10 μg sample of antibody diluted in 200 μL of lysate/washing buffer (25 mM Tris, pH 7.4, 150 mM NaCl, 1 mM EDTA, 1% Nonidet P-40, 5% glycerol, 0.25 mM phenylmethylsulfonyl fluoride) was added to 50 μL (1.5 mg) of 5% (w/v) bovine serum albumin (BSA)-blocked Dynabeads (Beyotime, China) and incubated at room temperature with rotation for 10 min. The supernatant was collected by centrifugation. The beads-Ab complex was washed twice in 200 μL of washing buffer, 200 μL of nuclear extract (1 mg) was then added and the mixture was incubated with rotation for 2 h at 4°C followed by centrifugation. Precipitates were washed five times with washing buffer, and the immunoprecipitated complexes were suspended in SDS sample buffer and analyzed by SDS-PAGE followed by Western blot analysis using the indicated antibodies.

### Electrophoretic Mobility Shift Assay (EMSA)

To test the binding of proteins to the regulatory sequences, electrophoretic mobility shift assay (EMSA) was performed according to the method of Kethidi et al. [35]. The labeled probes used in EMSA were the A element at nucleotide positions −64 to −39 (5′-CGAAAGTAAATACGTCAAAACTCGA-3′) and the B element at positions −87 to −63 (5′-AATGTGTAGATGTTTATTCTATC-3′) of the *fib-H* gene promoter [30]. The oligonucleotides were labeled using Cy3 from the 5′-end and then annealed to produce a double-stranded probe. DNA-binding reactions were carried out in a volume of 10 μL containing 10 μg of nuclear protein extracts or 1 μg of purified recombinant proteins and 2 μL of 5×binding buffer (Beyotime, China. Labeled probe (5 μM) was added after incubation for 20 min at 25°C and the incubation was continued for a further 25 min. For the competition experiments, unlabeled double-stranded probe was added to the reaction mixture at the same time as the labeled probe and mixtures were then loaded onto 5% (w/v) polyacrylamide gel and electrophoresed in 1×TBE buffer (45 mM Tris/borate, 1 mM EDTA, pH 8.3). After electrophoresis, the gel was scanned and photographed with a TYPHON scanner (Amersham).

### Statistical analysis

All data were statistically analyzed by independent sample *t*-test. Asterisks indicate significant differences (*, p<0.05; **, p<0.01).

## Results

### Bioinformatics analysis of the *Bmsage* gene

**Figure 1:**
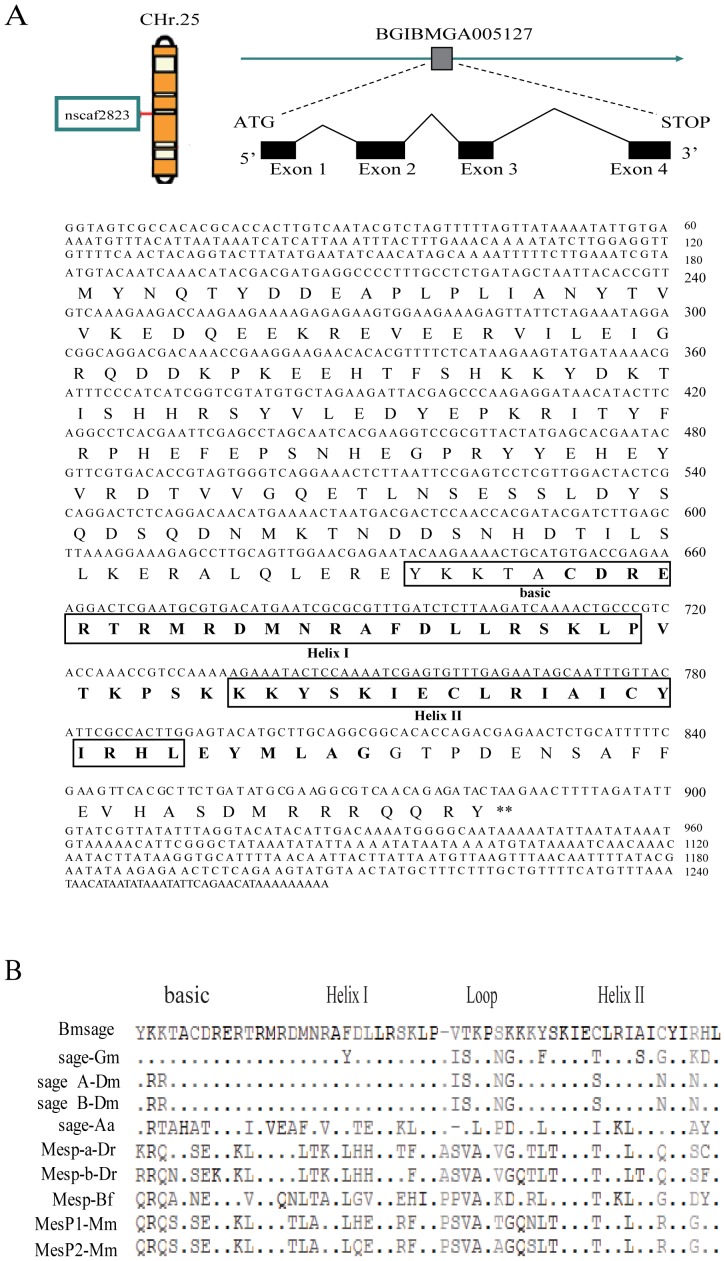
Bioinformatics analysis of *Bmsage*. **A.** The location of *Bombyx* sage in the silkworm genome (left), the structure (sketch map), and its nucleotide and deduced amino acid sequences. **B.** The bHLH motif of Bmsage is compared with those of other bHLH proteins, including Mesp subfamily of vertebrates, and Dmsage, Gmsage, Aasage of invertebrates, respectively. Dots indicate identical amino acids. Dashes represent gaps introduced to maximize the alignment.

According to the tissue chip data of the whole silkworm genome [36], we found a gene (ID: BGIBMGA005127) with a higher level of expression in the silk gland compared to other tissues (Fig. S1). It was located on chromosome 25 in silkworm precise mapping [37], which includes a single copy of a completed 705 bp coding frame, and consists of four exons spanning a 2.9 kb fragment in nscaf2823 (Fig. 1A). The predicted Bmsage protein consists of a single 234 amino acid with a bHLH domain (AA 157-210) (Fig. 1A). A blast search with the deduced amino acid sequence revealed several closely related Mesp subfamily of bHLH family members including vertebrate and invertebrate (Fig. 1B). The Bmsage bHLH domain showed 74%, 74%, 50%, 44.8%, 48.3% and 48.3% identity to those of Gmsage, Dmsage, Aasage, DrMesp, BfMesp and MmMesp, respectively (Fig. S2, Table S1). Based on this similarity and the phylogenetic tree, we concluded that Bmsage belongs to the Mesp-related subfamily of bHLH transcription factors.

### *Bmsage* expressed specifically in silk glands and closely relative to *fib-H* gene of silkworm

**Figure 2:**
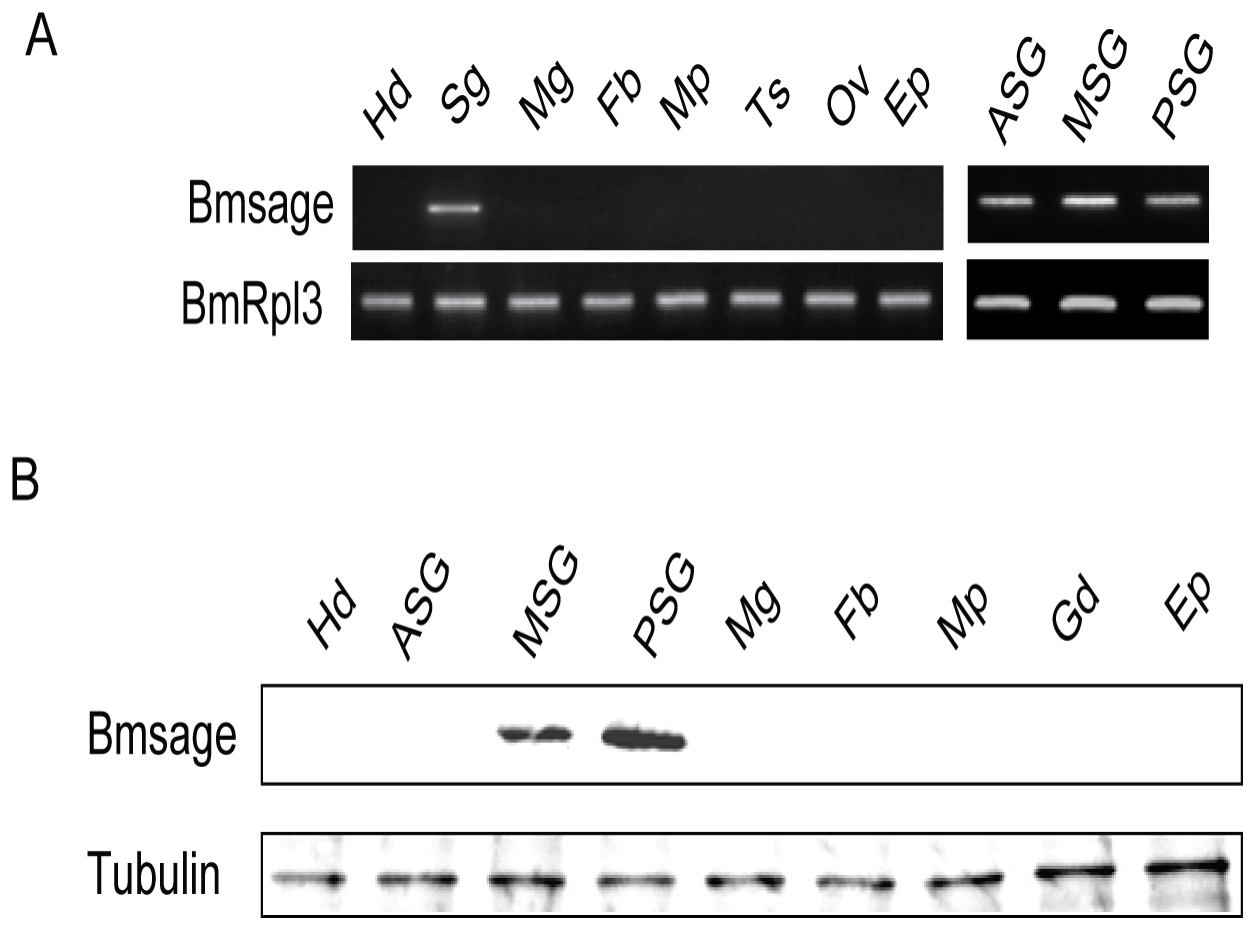
*Bmsage* expressed specifically in silk glands of *B. mori*. **A.** Expression level of *Bmsage* in different tissues of *B. mori* on day 3 of 5^th^ instar larvae by semiquantitative RT-PCR. *BmRpl3* expression is shown as a control. **B.** Protein level of Bmsage in different tissues of *B. mori* on day 3 of 5^th^ instar larvae. Tubulin is shown as a control. Different tissues are shown as: Hd, Head; ASG, Anterior silk gland; MSG, Middle silk gland; PSG, Posterior silk gland; Mg, Midgut; Fb, Fatbody; Mp, Malpighian; Gd, Gonad; Ep, Epidermis.

To verify *Bmsage* is expressed specifically in the silk glands, its level of expression was examined in different tissues of *B. mori* larvae on day 3 of the 5^th^ instar by semiquantitative RT-PCR (qPCR). As shown in Fig. 2, *Bmsage* is expressed only in silk glands from all tissues tested and distributed among the ASG, MSG and PSG cells (Fig. 2A). However, western blotting result showed that the Bmsage protein existed only in MSG and PSG cells, not in ASG cells (Fig. 2B).

**Figure 3:**
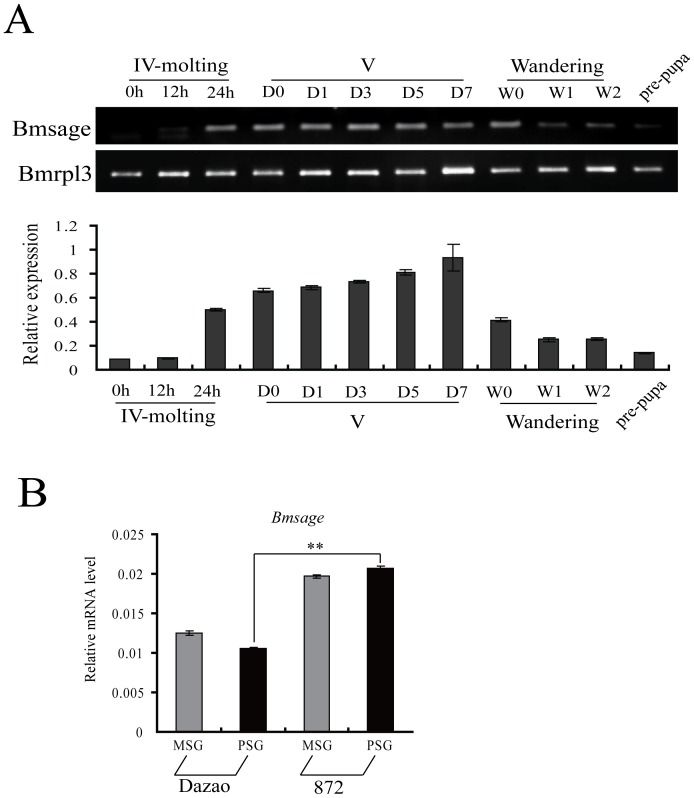
The relation of between *Bmsage* and *fib-H* in PSG of *B. mori*. **A.** Expression of *Bmsage* in different stages of *B. mori*. *BmRpl3* expression is shown as a control. Developmental stages are shown as: IV, 4^th^ instar; V, 5^th^ instar (0 day, 1 day, 3 day, 5 day, and 7 day); W, wandering (0 day, 1 day, 2 day) and prepupa. **B.** Expression of *Bmsage* in MSG and PSG by qPCR analysis. *BmRpl3* expression is shown as a control. Dz: Dazao, low silk strain; 872: high silk strain. The results are expressed as the means ± SD of three independent experiments. Asterisk indicate that the value is significantly different from control (** p<0.01).

On the basis of the results given above, we speculated that Bmsage might be involved in the regulation of silk protein genes. To explore the relation of *Bmsage* with *fib-H*, we used RT-PCR and qPCR to determine its expression level in the silk glands of 4^th^ instar molting, 5^th^ instar intermolt and wandering to pupation stages and in the production of different varieties of silk. The results showed that *Bmsage* is expressed in late 4^th^ instar molting and its level of expression increased with development in 5^th^ instar larvae and then declined in the wandering to pre-pupal stages (Fig. 3A). This expression pattern is almost consistent with that of *fib-H* gene in silkworm larvae (Fig. S3-A). In different silk-producing varieties, the cocoon shell weight and the cocoon shell rate of strain 872, which has high silk proteins in the silk glands, is higher compared to strain Dazao (Fig. S3-B). Furthermore, the expression of *fib-H* in 872 is up-regulated by 2.5-fold compared to strain Dazao on day 3 of the 5^th^ larval instar (Fig. S3-C). Interestingly, we found through the qPCR analysis *Bmsage* is up-regulated by 2.0-fold in the higher silk production strain 872 compared to the reference strain Dazao in the same period (Fig. 3B). Together, these results suggest the Bmsage might be involved in the regulation of *fib-H*.

### Bmsage interacts with SGF1

**Figure 4:**
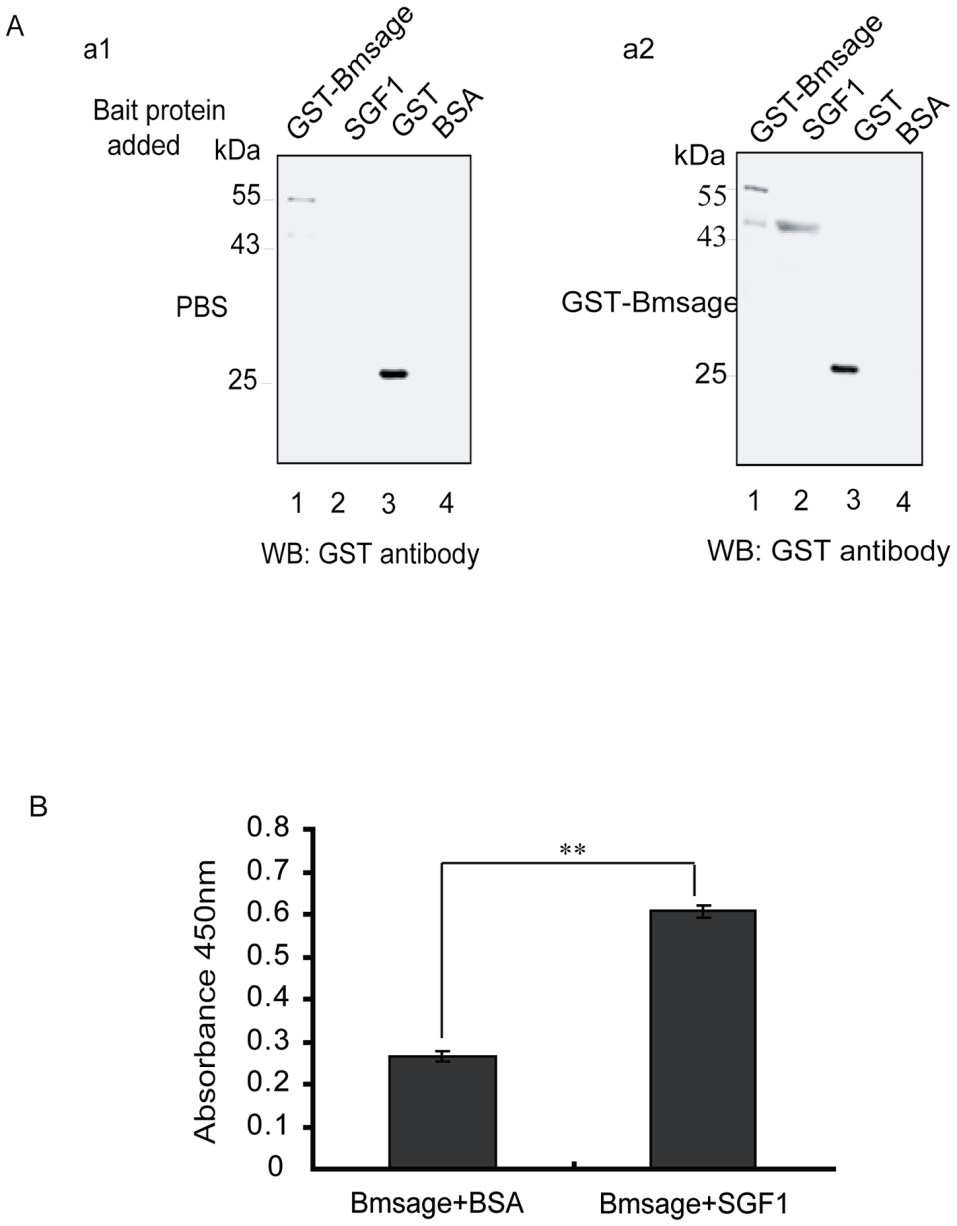
Bmsage protein interacts with SGF1 by far-western blot and ELISA analysis. **A.** Interaction analysis of Bmsage with SGF1. (a1) Purified Bmsage-GST as prey protein was incubated with PBS (as a control) and detected by anti-GST antibody. (a2) purified SGF1 as prey protein was incubated with purified Bmsage-GST (bait protein) and detected by anti-GST antibody. The purified GST protein was used as a positive control. BSA protein was used as a negative control. **B.** The binding of Bmsage and SGF1 was determined by ELISA. The BSA protein was used as a negative control. The results are expressed as the means ± SD of three independent experiments. Asterisk indicate that the value is significantly different from control (** p<0.01).

**Figure 5:**
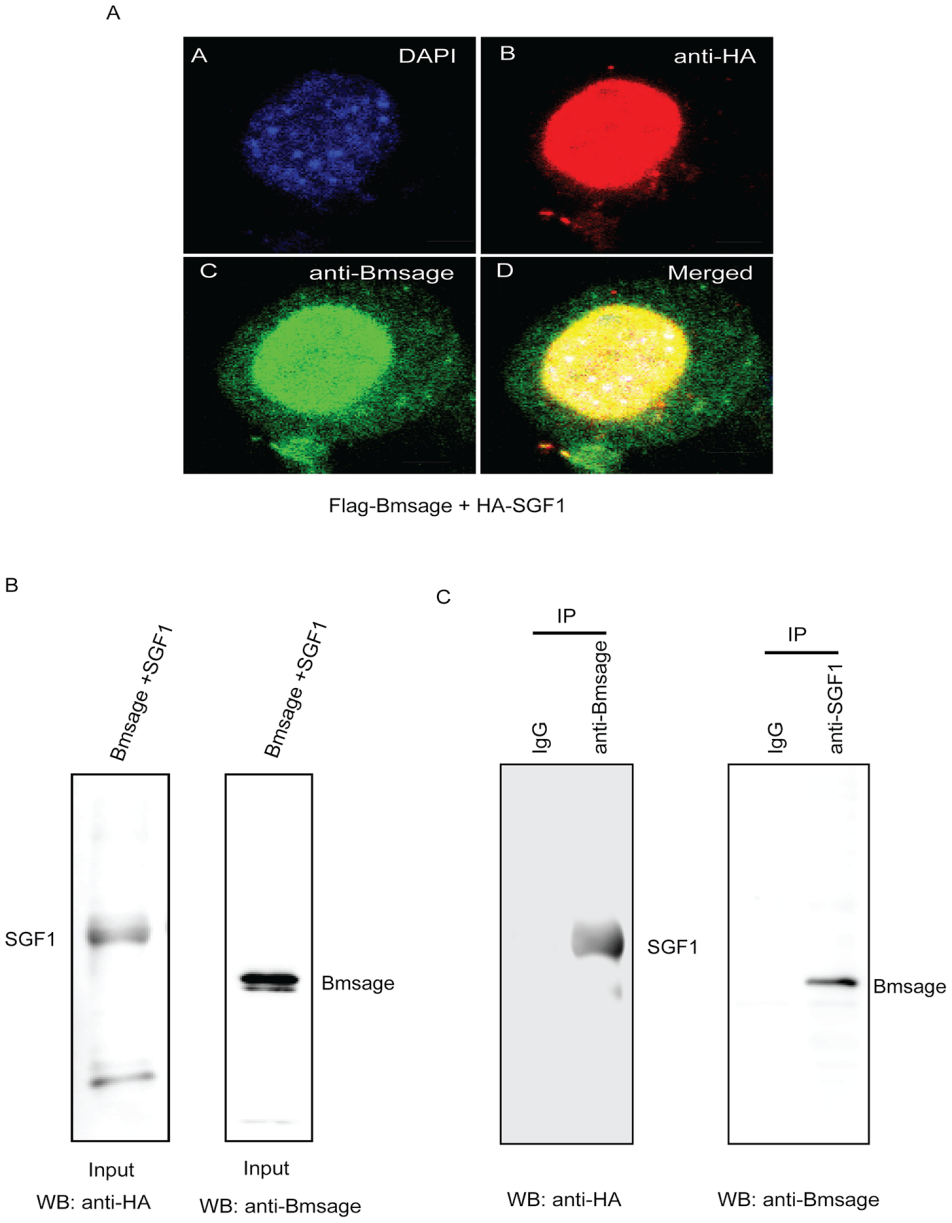
Immunohistochemical co-localization of Bmsage and SGF1 proteins and co-IP assay. **A.** Immunohistochemical co-localization of Bmsage and SGF1 in *Bm*E cells. HA-SFG1 and Flag-Bmsage were co-transfected into *Bm*E cells. Primary antibody [anti-HA monoclonal antibody mouse (Sigma) and anti-Bmsage antibody rabbit] was for 1 h, followed by incubation with the secondary antibody (anti- rabbit IgG FITC and anti-mouse Alexa 555) for 30 min, both at room temperature. The samples were mounted using a mounting medium containing DAPI and photographed using confocal microscopy (Japan). The scale bar shows 5 μm. **B.** Nuclear extracts from *Bm*E cells over-expressing Flag-Bmsage and HA-SGF1 were analyzed by Western blot (WB) using HA antibody (left) or Bmsage antibody (right). **C.** Nuclear extracts were immunoprecipitated (IP) with anti-Bmsage antibody or anti-SGF1 antibody followed by Western blot (WB) analysis using HA antibody (left) or Bmsage antibody (right).

As mentioned above, SGF1/fork head is the *Bombyx* homologue of the *Drosophila* fork head protein (Fkh), which co-regulates directly the expression of SG-specific genes with sage in the salivary gland. In *B. mori*, the SGF1 protein existed in MSG and PSG cells of day 3 of 5^th^ instar larvae, as found for the Bmsage protein (Fig. S4). To determine whether Bmsage and SGF1 can interact directly in vitro, a far-western blot assay was used with purified full-length His-SGF1 and Bmsage-GST fusion proteins. The results showed that Bmsage could bind to SGF1 on the PVDF membrane (∼41 kDa) (Fig. 4A-a2, lane 2), but the control could not (Fig. 4A-a1, lane 2). An ELISA binding assay showed that absorbance at 450 nm was significantly different between Bmsage/SGF1 and Bmsage/BSA (Fig. 4B), suggesting Bmsage is able to bind to SGF1 in vitro. Based on the data presented above, we asked whether SGF1 and Bmsage can interact in vivo. To address the issue, we expressed HA-tagged SGF1 and Flag-tagged Bmsage in *Bm*E cells using a BmA4 promoter-driven construct. The results of immunohistochemical analysis showed that SGF1 and Bmsage were localized in the nucleus (Fig. S5) and colocalized within the same cells (Fig. 4A, A–D). Nuclear extracts from *Bm*E cells over-expressing Flag-sage and HA-SGF1 were immunoprecipitated with anti-Bmsage antibody or anti-SGF1 antibody followed by western blot analysis using HA antibody or Bmsage antibody. As shown in Fig. 5, the SGF1 protein was present in an anti-Bmsage immunoprecipitate (Fig. 5C, left). *Vice versa*, Bmsage protein was present in an anti-SGF1 immunoprecipitate (Fig. 5C, right). These results indicated the Bmsage protein was able to interact with the SGF1 protein to form a complex in the cell nucleus.

### Complex of Bmsage and SGF1 binds to the A and B elements in the *fib-H* gene promoter and up-regulates expression of the *fib-H* gene

**Figure 6:**
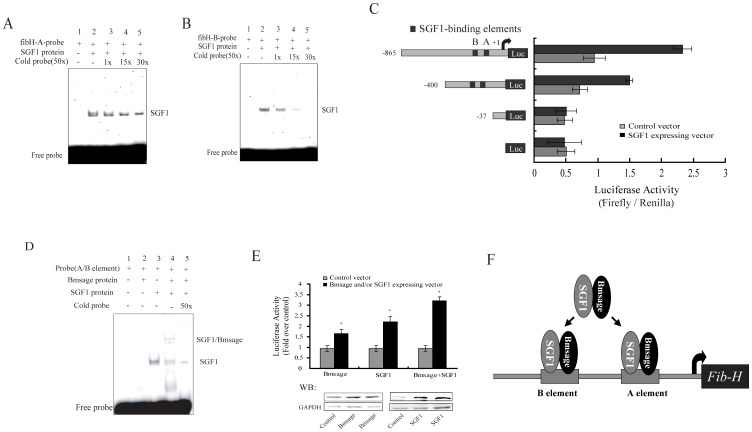
Complex of Bmsage and SGF1 proteins binds to A and B elements and model for regulation of *fibroin* H-chain gene in *B. mori*. **A–B.** SGF1 protein binds to the A and B elements in vitro by EMSA. **C.** Three different 5′ truncated fragments from the promoter of *fib-H* were created by PCR amplification and transfected into *Bm*E cells for luciferase expression analysis. **D.** The recombinant Bmsage and SGF1 proteins binds to the A and B elements in vitro by EMSA. **E.** Effect of recombinant Bmsage and SGF1 proteins on the expression of the reporter luciferase under the control of the *fib-H* promoter. One microgramme of the reporter plasmid DNA with 1 μg 1180-A4/Flag-Bmsage and/or 1180-A4/HA-SGF1 was transfected or co-transfected into *Bm*E cells. The cells were cultured for an additional 48 h and harvested for luciferase expression analysis. 1180-A4/EGFP plasmid was as a control. The relative luciferase activity was presented as a ratio of the firefly luciferase activity to the *Renilla* luciferase activity. The result was repeated three times independently and the results of the average expression level was expressed as mean±SE. Asterisk indicate that the value is significantly different from control (* p<0.05). **F.** A model for regulation of *fibroin* H-chain gene in *B. mori*. The nuclear bHLH transcription factor Bmsage interacts with fork head transcription factor SGF1, and this complex binds to the A and B elements in the promoter of *fib-H* involved in the expression of *fibroin* H-chain gene in *B. mori* PSG cells.

Earlier studies showed there are two regions (the A element between positions −64 and −39 and the B element between −87 and −63) in the *fib-H* promoter and SGF1 can bind to the A and B elements in vitro [4]. However, evidence of this interaction in vivo is not available. In order to study the functional role of SGF1 in transcription, the SGF1 protein and different 5′ truncated fragments of the *fib-H* regulatory sequence were prepared. Unsurprisingly, recombinant SGF1 protein alone could bind directly to the A and B elements of the *fib-H* promoter and the binding was inhibited competitively when the amount of cold probe was increased gradually (Fig. 6A, B). In addition, the luciferase expression activity of these constructs was assessed by transfecting the *Bm*E cell line. Expression activity was increased dramatically compared to the control by over-expressing SGF1. However, when the region between positions −400 and −37 containing A and B elements was deleted, the expression activity was not significantly different from the control (Fig. 6C). These results indicated that the A and B elements were involved in the regulation of *fib-H* expression in the *Bm*E cell line. Whether Bmsage works with SGF1 involved in the regulation of *fib-H* by the elements, however, remains poorly understood. Thus, to determine whether a complex of Bmsage and SGF1 proteins binds to the A and B elements of the *fib-H* promoter directly, purified Bmsage and SGF1 proteins were incubated with the A or B element DNA probe in vitro and then analyzed by EMSA. The recombinant Bmsage protein alone was not able to bind to the A and B elements probe (Fig. 6D, lane 2), but the complex of Bmsage and SGF1 protein bound to the A and B regions of the *fib-H* promoter and retarded the mobility of this complex compared to recombinant SGF1 protein (Fig. 6D, lane 3–4), and the binding was partially inhibited by a 50-fold excess of the cold probe (Fig. 6D, lane 5). Therefore, we hypothesized Bmsage could be involved in the regulation of *fib-H* by making contact with SGF1. To test this, we used the luciferase reporter assay to examine whether Bmsage and SGF1 regulate the *fib-H* promoter activity. As shown in Fig. 6, the level of expression of the luciferase gene under the control of the *fib-H* promoter increased about 3.0-fold compared to the control in cells by over-expressing Bmsage and SGF1 (Fig. 6E). Together, these results suggest that the Bmsage protein is able to interact with the SGF1 protein to form a complex and regulate expression of the *fib-H* gene.

## Discussion

In the present study, by scanning the microarray database of the silkworm *B. mori* we identified an Mesp subfamily bHLH transcription factor that shares 74% amino acid sequence identity with sage from *D. melanogaster* [38]. This transcription factor has been found in vertebrates and invertebrates and has important roles in cell type-specific gene expression and cell fate determination; e.g. *D. rerio* and *D. melanogaster* [24], [39]. Here, *Bmsage* was shown to be expressed only in the silk glands of *B. mori* and has a low level of expression in the 4^th^ instar molting stages, increasing gradually to a high level in feeding stages of the 5^th^ instar (Fig. 2 and Fig. 3). The expression patterns of *Bmsage* and silk protein-encoding genes are similar. Previous studies showed several transcription factors were involved in transcriptional regulation of the silk genes in the silk glands of *B. mori*. Besides binding to the proximal upstream elements of both the *fibroin* and *sericin-1* genes, the SGF1/fork head acts as a crucial transcription activator of the *P25* gene in PSG cells by binding to the promoter element [40]. SGF-2, which is a 1.1 MDa heteromeric complex containing Awh, Ldb, Lcaf and fibrohexamerin proteins [10], bound three distal regions of the upstream modulator of the *fibroin* gene, which is composed of AT-rich repeats [7]. SGF-3, which was identified as POU-M1 [12], regulates the *sericin-1* gene negatively [41]. The *Bmsage* gene, which has a distinct spatial specificity in *B. mori,* also play an important role in the silk glands.

The members of the bHLH superfamily have been classified into various families: the bHLH-Per-Arnt-Sim domain, the Hairy-Enhancer of split (HES), the Myc/Upstream Transcription Factor (USF), Atonal, Mesp, Hand, p48, NeuroD/Neurogenin family, Shout and Achaete-scute (AS-C) [42]. The major finding of this work was the discovery that Bmsage, which belongs to the Mesp subfamily (Fig. 1C), existing specifically in the silk glands of *B. mori*. A superfamily of transcription factors containing bHLH mainly activates expression of the target via bind to E-box (CANNTG) sequence [43]. The level of expression of the luciferase gene under the control of the *fib-H* promoter (-865 - +1bp) increased by 1.5-fold over the control in cells through over-expressing Bmsage (Fig. 6E). However, we have not found a specific bind site (E-box sequence) in the proximal region of the 5′ flanking sequence of the *fibroin* gene. It is possible that Bmsage interacts with endogenous SGF1 protein and is involved in the regulation of the *fib-H* gene.

The silk glands of *B. mori* produce vast amounts of several silk proteins secreted mainly in the MSG and PSG cells [5]. The genes encoding these silk proteins are expressed actively in the feeding stages but are repressed during the molting stages [44]. Among them, the *fibroin* genes are highly expressed in the PSG cells but are repressed in MSG cells. The Bmsage protein was detected only in the MSG and PSG cells, not in ASG cells (Fig. 2B), indicating Bmsage might be correlated with synthesis of silk proteins in *B. mori* MSG and PSG cells. In *D. melanogaster*, the salivary glands are specialized also for the massive production of several tissue-specific secretory proteins; sage is a salivary gland-specific bHLH protein that works with Fkh to regulate expression of *SG2* directly [24]. In *B. mori*, SGF1 is a fork head factor, which is a homologue of the *Drosophila* fork head protein, and is present in the silk gland nuclei during the whole course of larval life [45]. Furthermore, the *B. mori* silk glands are regarded as organs homologous with the *D. melanogaster* salivary glands [25]. Previous studies showed that SGF1 was able to bind to the A and B elements of the *fibroin* gene promoter in vitro [4], but we, for the first time, provided direct evidence that SGF1 interacted with Bmsage and regulated expression of the *fibroin* gene by binding to the −400 and −37 region containing A and B elements (Fig. 6). However, we found that the expression activity was reduced when the region between −865 and −400 of the *fib-H* gene regulatory sequence was deleted. This result indicated the region between −865 and −400 contained *cis*-regulatory elements (CREs) possibly involved in the regulation of *fib-H* expression. In addition, the 5′ flanking sequence of the *B. mori fibroin* gene, which is known to be required for a maximal level of transcription in vitro, contains five regions (A to E) that bind protein factors from the PSG extract [4]. Besides the proximal A and B regions, the promoter of *fib-H* gene contains three distal regions (C, D and E) which bind one posterior silk gland factor (SGF-2) and two ubiquitous factors (SGF-3 and -4). These factors have an important role in the expression of silk protein genes, and the clustering of the C, D and E regions in the *fibroin* gene promoter might be necessary to create a high-affinity site for these silk gland proteins [7], [10], [19].

On the basis of data in the literature and our own results, we propose a new model for the regulation of the *fib-H* gene (Fig. 6F). The nuclear bHLH transcription factor Bmsage interacts with fork head transcription factor SGF1 and this complex binds to the A and B elements in the promoter of *fib*-H gene involved in expression of the *fib-H* gene in *B. mori* PSG cells. This is the first report that the transcription factor containing a bHLH domain is expressed specifically in silk glands and is involved in the regulation of the expression of *fib-H* gene in *B. mori*. Thus, our findings provide a new insight into the regulation of other genes encoding *B. mori* silk proteins.

## Supporting Information

Figure S1 **Expression of** ***Bmsage*** **in multiple silkworm tissues on day 3 of the fifth instars based on microarray database.**
(TIF)

Figure S2 **A phylogenetic tree of Bmsage.** A phylogenetic tree of bHLH transcription factors is constructed using the MEGA5 program with the neighbor-joining algorithm. GenBank accession numbers were shown in Table S2.
(TIF)

Figure S3 **Cocoon shell rate of Dazao and 872.** **A.** Expression of *fib-H* in different stages of *B. mori*. *BmRpl3* expression is shown as a control. Developmental stages are shown as: IV-molting: 4^th^ instar molting, V: 5^th^ instar feeding stages (1 day, 3 day, 5 day, and 7 day), and prepupa. **B.** The cocoon picture (left) and the cocoon shell rate (right) of 872 and Dazao. The scale bar shows 1 cm. The experiment were set as three groups independently and each group has five individuals. The result was showed as mean±SE. **C.** Expression of *fib-H* in MSG and PSG by qPCR analysis. *BmRpl3* expression is shown as a control. Dz: Dazao, low silk strain; 872: high silk strain. The results are expressed as the means ± SD of three independent experiments. Asterisk indicate that the value is significantly different from control (** p<0.01).
(TIF)

Figure S4 **Protein level of SGF1 in different tissues of** ***B. mori*** **on day 3 of 5^th^ instar larvae by western blot analysis.** Tubulin is shown as a control. Different tissues are shown as: ASG, Anterior silk gland; MSG, Middle silk gland; PSG, Posterior silk gland; Gd, Gonad; Mp, Malpighian; Mg, Midgut; Fb, Fatbody; Ep, Epidermis; Hd, Head.
(TIF)

Figure S5 **Immunohistochemical localization of Bmsage and SGF1 proteins in** ***Bm*** **E cells.** HA-SFG1 and Flag-Bmsage were transfected into *Bm*E cells. Primary antibody [anti-HA monoclonal antibody mouse (Sigma) or anti-Flag monoclonal antibody M2 mouse (Sigma)] was for 1 h, followed by incubation with the secondary antibody (anti-mouse Alexa 555) for 30 min, both at room temperature. The samples were mounted using a mounting medium containing DAPI and photographed using confocal microscopy (Japan). The scale bar shows 5 μm.
(TIF)

Table S1 **Name of gene and accession number for phylogenetic analysis from NCBI.**
(DOCX)

Table S2 **Primer sequences used in this study.**
(DOCX)
