# Supplementary figures and images for: Basic Helix-Loop-Helix Transcription Factor Bmsage Is Involved in Regulation of fibroin H-chain Gene via Interaction with SGF1 in Bombyx mori
Source: PLoS One. 2014 Apr 16;9(4):e94091. doi: 10.1371/journal.pone.0094091 (PMC3989216; doi:10.1371/journal.pone.0094091)

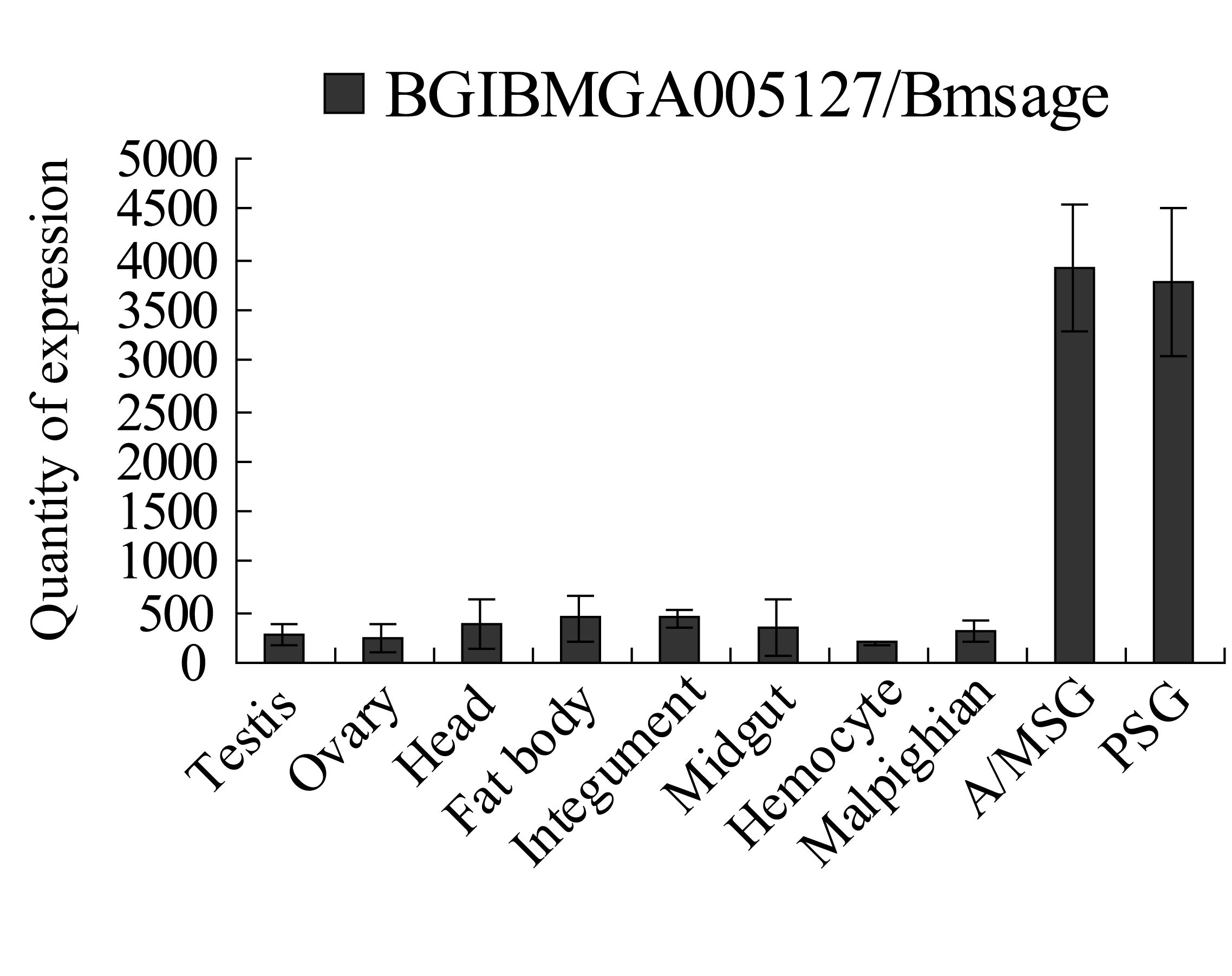

Supplement: Figure S1 — Expression of Bmsage in multiple silkworm tissues on day 3 of the fifth instars based on microarray database. (TIF) [file pone.0094091.s001.tif]

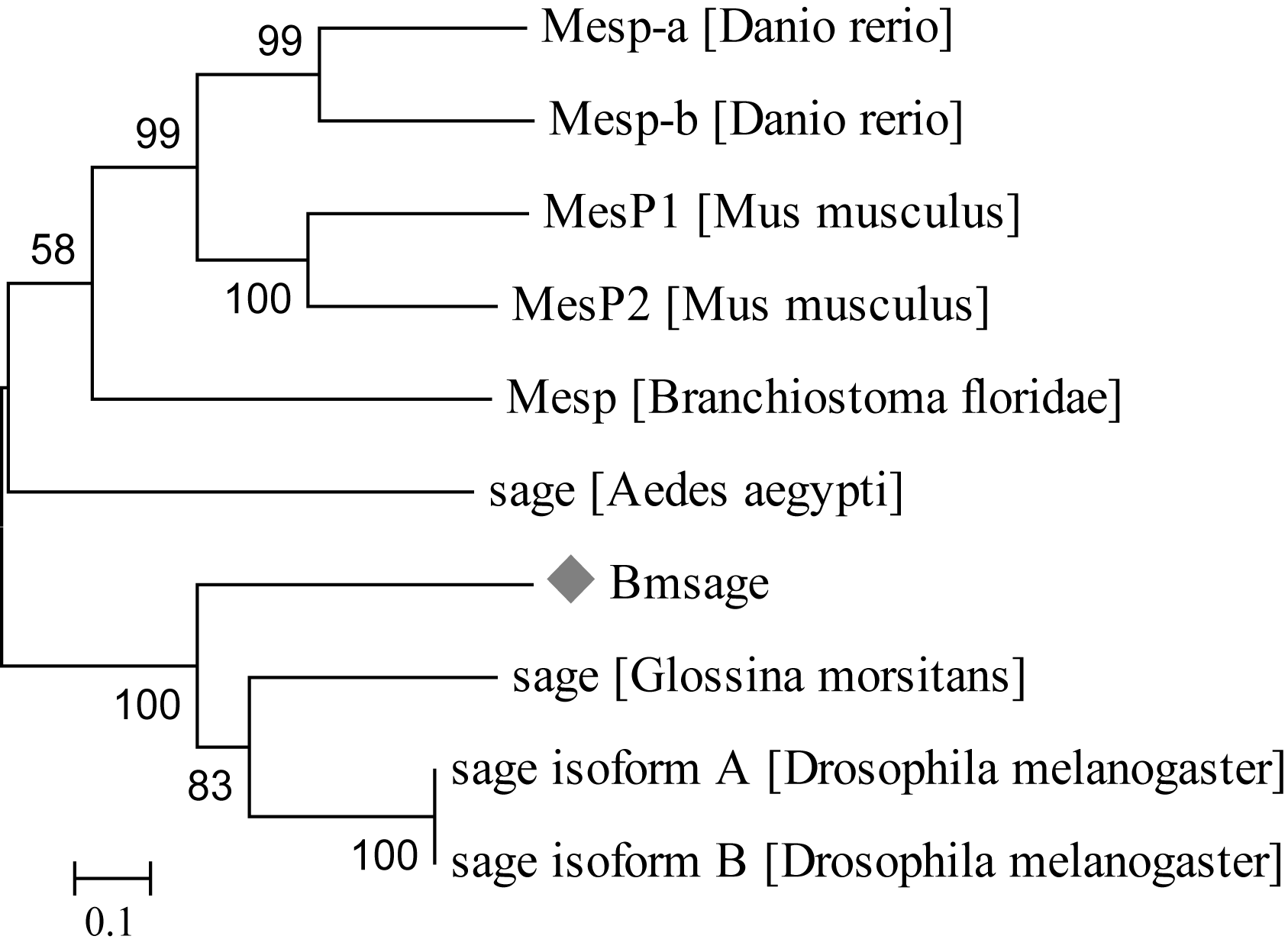

Supplement: Figure S2 — A phylogenetic tree of Bmsage. A phylogenetic tree of bHLH transcription factors is constructed using the MEGA5 program with the neighbor-joining algorithm. GenBank accession numbers were shown in Table S2. (TIF) [file pone.0094091.s002.tif]

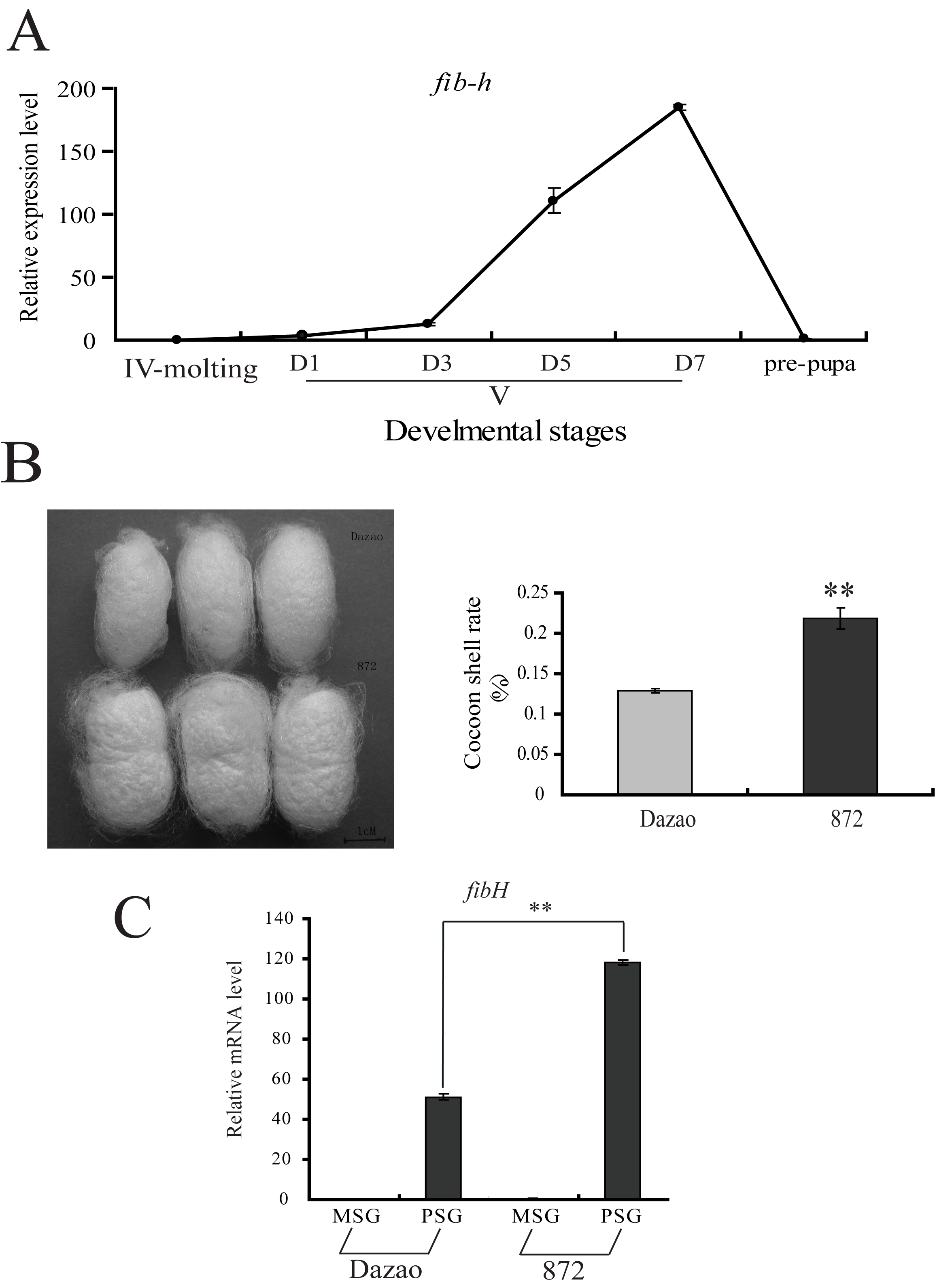

Supplement: Figure S3 — Cocoon shell rate of Dazao and 872. A. Expression of fib-H in different stages of B. mori. BmRpl3 expression is shown as a control. Developmental stages are shown as: IV-molting: 4th instar molting, V: 5th instar feeding stages (1 day, 3 day, 5 day, and 7 day), and prepupa. B. The cocoon picture (left) and the cocoon shell rate (right) of 872 and Dazao. The scale bar shows 1 cm. The experiment were set as three groups independently and each group has five individuals. The result was showed as mean±SE. C. Expression of fib-H in MSG and PSG by qPCR analysis. BmRpl3 expression is shown as a control. Dz: Dazao, low silk strain; 872: high silk strain. The results are expressed as the means ± SD of three independent experiments. Asterisk indicate that the value is significantly different from control (** p<0.01). (TIF) [file pone.0094091.s003.tif]

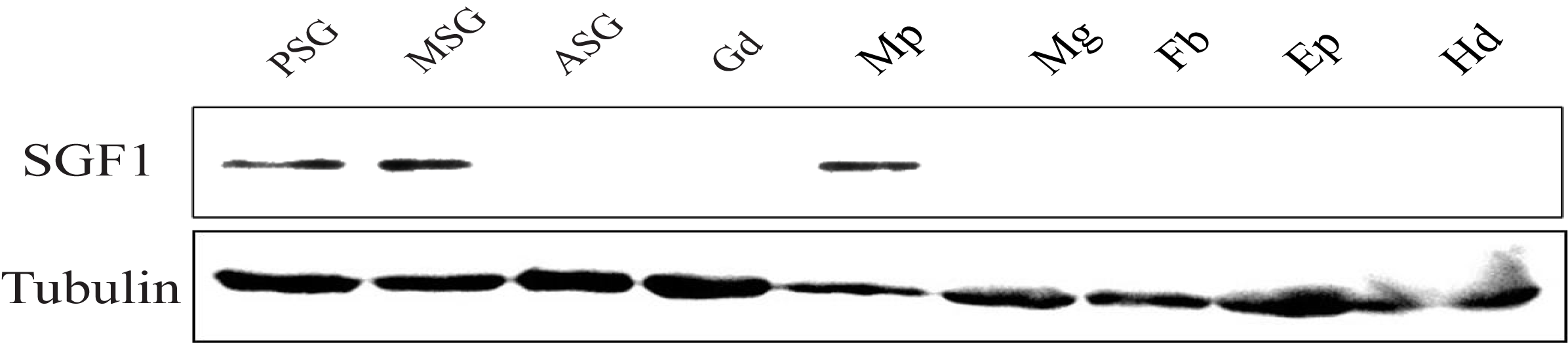

Supplement: Figure S4 — Protein level of SGF1 in different tissues of B. mori on day 3 of 5th instar larvae by western blot analysis. Tubulin is shown as a control. Different tissues are shown as: ASG, Anterior silk gland; MSG, Middle silk gland; PSG, Posterior silk gland; Gd, Gonad; Mp, Malpighian; Mg, Midgut; Fb, Fatbody; Ep, Epidermis; Hd, Head. (TIF) [file pone.0094091.s004.tif]

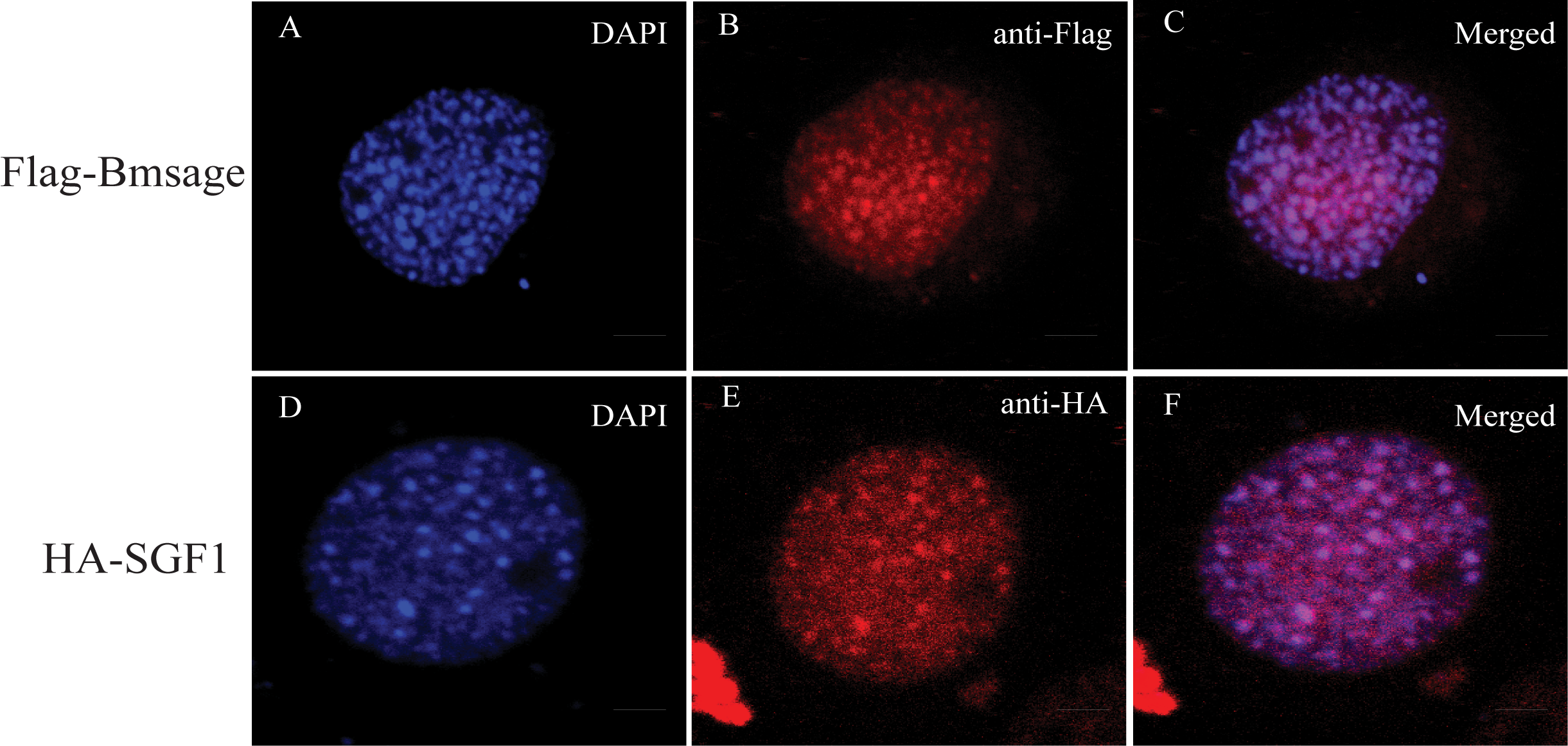

Supplement: Figure S5 — Immunohistochemical localization of Bmsage and SGF1 proteins in Bm E cells. HA-SFG1 and Flag-Bmsage were transfected into BmE cells. Primary antibody [anti-HA monoclonal antibody mouse (Sigma) or anti-Flag monoclonal antibody M2 mouse (Sigma)] was for 1 h, followed by incubation with the secondary antibody (anti-mouse Alexa 555) for 30 min, both at room temperature. The samples were mounted using a mounting medium containing DAPI and photographed using confocal microscopy (Japan). The scale bar shows 5 μm. (TIF) [file pone.0094091.s005.tif]
