# Supplementary material for: Basic Helix-Loop-Helix Transcription Factor Bmsage Is Involved in Regulation of fibroin H-chain Gene via Interaction with SGF1 in Bombyx mori
Source: PLoS One. 2014 Apr 16;9(4):e94091. doi: 10.1371/journal.pone.0094091 (PMC3989216; doi:10.1371/journal.pone.0094091)
Supplement: Table S1 — Name of gene and accession number for phylogenetic analysis from NCBI. (DOCX) [file pone.0094091.s006.docx]

| Name | Species | Accession number |
| --- | --- | --- |
| sage | *Bombyx mori* | AKC820642 |
| sage isoform A | *Drosophila melanogaster* | NP_524287.1 |
| sage isoform B | *Drosophila melanogaster* | NP_731326.1 |
| sage | *Aedes aegypti* | XP_001649518.1 |
| Mesp-a | *Danio rerio* | AB037939 |
| Mesp-b | *Danio rerio* | AB037940 |
| sage | *Glossina morsitans* | ADD18822.1 |
| Mesp | *Branchiostoma floridae* | ABD57444.1 |
| MesP1 | *Mus musculus* | BAA12041.1 |
| MesP2 | *Mus musculus* | AAB51199.1 |
