# Supplementary material for: Basic Helix-Loop-Helix Transcription Factor Bmsage Is Involved in Regulation of fibroin H-chain Gene via Interaction with SGF1 in Bombyx mori
Source: PLoS One. 2014 Apr 16;9(4):e94091. doi: 10.1371/journal.pone.0094091 (PMC3989216; doi:10.1371/journal.pone.0094091)
Supplement: Table S2 — Primer sequences used in this study. (DOCX) [file pone.0094091.s007.docx]

| Assay | Gene | Primer sequences (5′−3′) |
| --- | --- | --- |
| RT-PCR | *Bmsage* | F:ATGTACAATCAAACATAC |
|  |  | R: TATCTCTGTTGACGC |
| RT-PCR | *BmRpl3* | F:TCGTCATCGTGGTAAGGTCAA |
|  |  | R:TTTGTATCCTTTGCCCTTGGT |
| qRT-PCR | *Bmsage* | F: AGCAATCACGAAGGTCCGC |
|  |  | R: CGTATCGTGGTTGGAGTCGT |
| qRT-PCR | *fib-H* | F:TATCCAGGACGAAGTAAGAAACAA |
|  |  | R: TCTGTGTCATCTGCTTCATCTCG |
| qRT-PCR | *BmRpl3* | F: TTCGTACTGGCTCTTCTCGT |
|  |  | R: CAAAGTTGATAGCAATTCCCT |
